# The intraoperative use of a calliper predicts leg length and offset after total hip arthroplasty. Component subsidence influences the leg length

**DOI:** 10.1186/s13018-021-02559-3

**Published:** 2021-07-03

**Authors:** Maliha Fansur, Nagib A. Yurdi, Reinhard Stoewe

**Affiliations:** 1 Department of Radiology, Lincoln County Hospital, Lincoln, LN2 5QY UK; 2 Department of Orthopedics, Healthpoint Hospital, Abu Dhabi, UAE; 3 Department of Orthopedics, Sønderborg Hospital, 6400 Sønderborg, Denmark

**Keywords:** Hip arthroplasty, Leg length, Offset, Calliper, Subsidence

## Abstract

**Background:**

The purpose of total hip arthroplasty (THA) post-surgery and proper physiotherapy is positive recovery for the patient. Consideration is given to hip replacement biomechanics by ensuring no discrepancies in limb length (LL) and a stable prosthesis. Therefore, the patient must have proper preoperative planning and communication and a clear understanding of what to expect.

**Methods:**

A prospective series of 59 THA operated by a single surgeon via Hardinge approach was studied, using an intraoperative calliper (CAL) to predict the change of LL and offset. We compared the results of the intraoperative changes before and after THA implantation with the reference of these values on anteroposterior x-ray pelvis. The importance of leg length balance and a good offset restoration is questioned, and the effect of component subsidence on leg length is considered.

**Results:**

The average preoperative leg length discrepancy was −6.0 mm, postoperatively +3.6 mm. There was a strong correlation between the CAL measurements and the values on the x-ray (LL, r=0.873, p<0.01; offset, r=0.542, p<0.01). Reliability is better for limb length than for offset. These results are comparable within the literature and the statistical results from other studies reviewed. In addition, we evaluate the importance of subsidence of the prosthesis components for long-term results.

**Conclusion:**

The intraoperative use of CAL gives excellent results in predicting the final LL and offset after THA. Considering subsidence of prosthesis components, a target zone around +5 mm might be more suitable for leg length directly postoperatively. Moreover, surgeons must discuss the topic of leg length discrepancy (LLD) intensively with the patient pre-operatively.

**Level of evidence:**

Level 4, prospective cohort study

## Introduction

Since its introduction more than 70 years ago, total hip arthroplasty (THA) is one of the most successful and frequently performed procedures in orthopedic surgery. After total hip arthroplasty, perceived residual leg length discrepancy (LLD) can cause limping and patient dissatisfaction [1]. Despite of the introduction of modern methods to improve the results, such as templating, computer navigation, intraoperative imaging, and minimal invasive approaches, orthopedic surgeons still feel uncertainty about the ideal postoperative leg length and offset after THA. Recently, also as response to patient’s dissatisfaction and threatening litigation [2], there have been an increasing number of studies to suggest improvements concerning this aspect by using intraoperative tests, calibration gauges, and other devices to improve functional outcome and reduce possible complications. One difficulty when approaching this problem is the interference with other phenomena observed in THA surgery, such as hip instability, gait disorders, infection, fractures, and subsidence of prosthetic components.

While the use of an intraoperative calibration gauge is widespread in some countries, e.g., Norway, there are other countries where it is hardly used, e.g., Germany and the UAE. Therefore, the authors decided it was valuable to present the results from this study suggesting the value of an intraoperative calibration gauge to make THA more predictable. The hypothesis of this study is that the use of an intraoperative calliper can predict the postoperative leg length and offset, and therefore improve the outcome of THA operations. The first section presents the results related to postoperative leg length and offset using an intraoperative calliper (CAL). The values from anterior-posterior (AP) x-ray of the pelvis with a calibration ball are used as a reference. Utilizing this measurement method, the target zone for intraoperative leg length measurement is discussed with respect to other interfering factors.

## Materials and methods

This study was completed at Healthpoint Hospital in the city of Abu Dhabi, UAE. Healthpoint Hospital is a semi-public hospital in the capital of the UAE functioning as a local hospital and a reference hospital for the whole country. This clinical study was registered and authorized by the institutional research ethics committee REC016.

### Method

This is a retrospective study of prospectively collected data where 54 patients and 59 hips were reviewed. All THA operations were performed by the senior author RS between 2018 and 2020. All patients signed a consent form to participate in this study. All patients with stable and reproducible hip conditions on the side to be operated, including primary and revision surgery, were included in this study. Patients with acute proximal femoral fractures and dislocated dysplasia hips were excluded. In a reflection of the population diversity of the UAE, the clinical group came from 21 different national backgrounds. The patient demographic characteristics are shown in Table 1.

**Table 1:** Patient demographic characteristics and procedure data

Age mean	51 (17–74)
Gender (M/F)	30/24
Operated hip (L/R)	30/29
BMI mean	30 (20–47)
Discharge post-OP day mean	4.8 (1–10)
Primary THA/revision THA	56/3
Femoral component (uncemented/cemented)	55/4
Acetabular component (uncemented/cemented)	57/2

Preoperatively, all patients were examined clinically. In case of suspected LLD, an examination of the patient standing, using lift blocks under the short leg, and visually examining the level pelvis was completed. In this study, the gold standard and reference to measure preoperative LLD was the anterior-posterior (AP) weight-bearing x-ray of the pelvis [3]. Other radiological examinations to further examine LLD preoperatively, such as x-ray long-film standing, scanograms, computerized digital radiographs, and CT [4, 5], were utilized in exceptional circumstances on some patients. The results of these additional examinations performed were not included in this study.

### Surgical technique

**Fig. 1:**
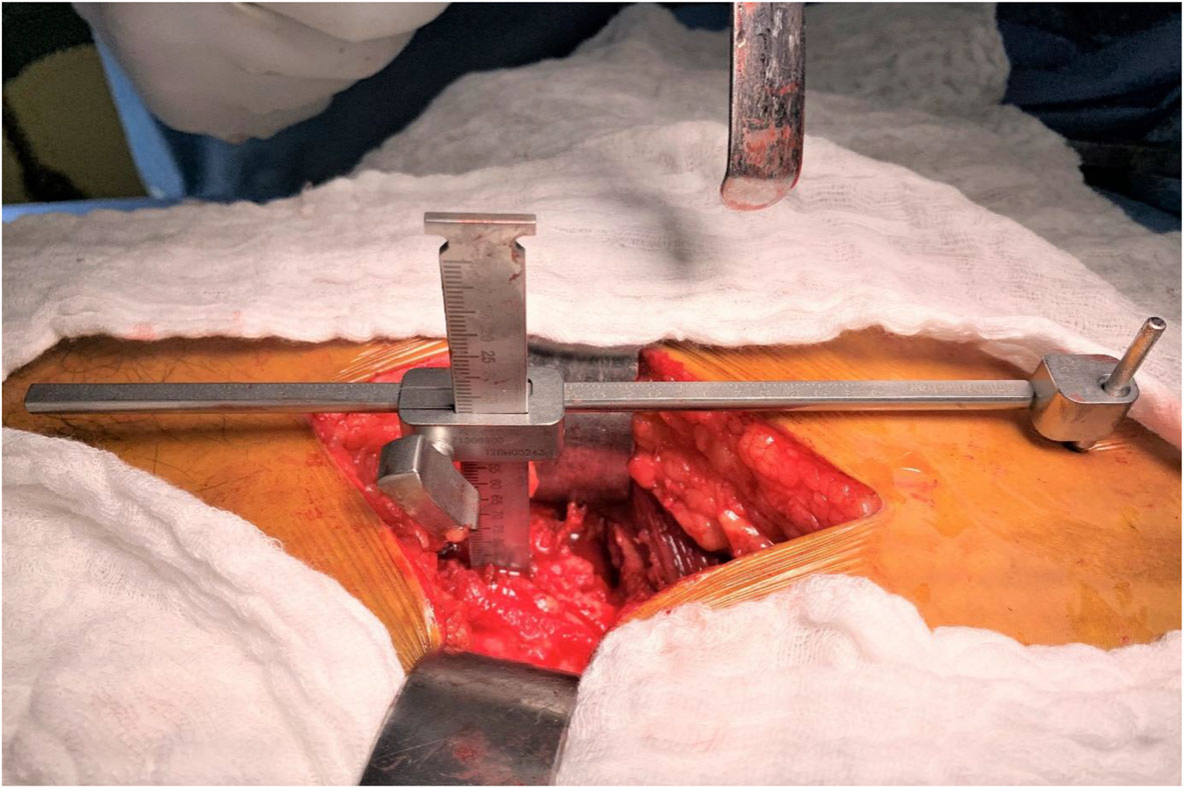
The calliper set in place before the dislocation of the hip operating a right hip patient lateral supine

All surgeries were performed via a direct lateral Hardinge approach, patient in lateral decubitus. All prosthesis was distributed by DePuy Synthes. The CAL was supplied by Smith & Nephew, Memphis, TN, USA. Its proper use and possible mistakes while using it are described well in the literature [6, 7]. The reference pin for the use of CAL was positioned into the pelvis in line with the skin incision close to the iliac crest through an additional 3-mm skin incision. Before the dislocation of the hip, the most lateral point of the greater trochanter was marked with diathermy on the bone and the surrounding soft tissue. After attaching the free arm of CAL to the reference pin, the tip was gently led onto the mark on the greater trochanter without creating any tension in the CAL. Figure 1 shows the calliper set in place before the dislocation of the hip operating a right hip patient lateral supine.

In this position, the first set of data was recorded and documented. The second set of data was recorded likewise when the trial implants were in place. If there was a substantial aberration of this data from expected values, the reason, such as cup malpositioning or severe varus/valgus malalignment of the stem, was found and corrected. At this stage, and at the end of the operation before wound closure, also different clinical tests were done to check the stability of the hip and to look for any possible impingement, the priority being to achieve a stable hip [8]. Based on the second set of data, the final implants were chosen (neck length, standard or high offset of the stem, stem size) and set in place. The third set of data was the values after the last reposition of the hip with the final components in place. To avoid deviation due to positioning, this third set of data was taken with a particular focus on maintaining the operative limb in the same position while taking the first set concerning flexion and abduction. This process is also commonly described in the literature [7]. The first and third set of data were used in this study. The change of leg length can be calculated immediately. This is not the case for the femoral offset, which per definition is the distance from the center of rotation of the femoral head to a line bisecting the long axis of the femur [9]. We were aware that by using CAL we measured a change in a value closely related to global offset, which by definition is the sum of the femoral offset and the lateralization of the hip joint center of rotation (acetabular offset) [9, 10].

### Radiological analysis

**Fig. 2:**
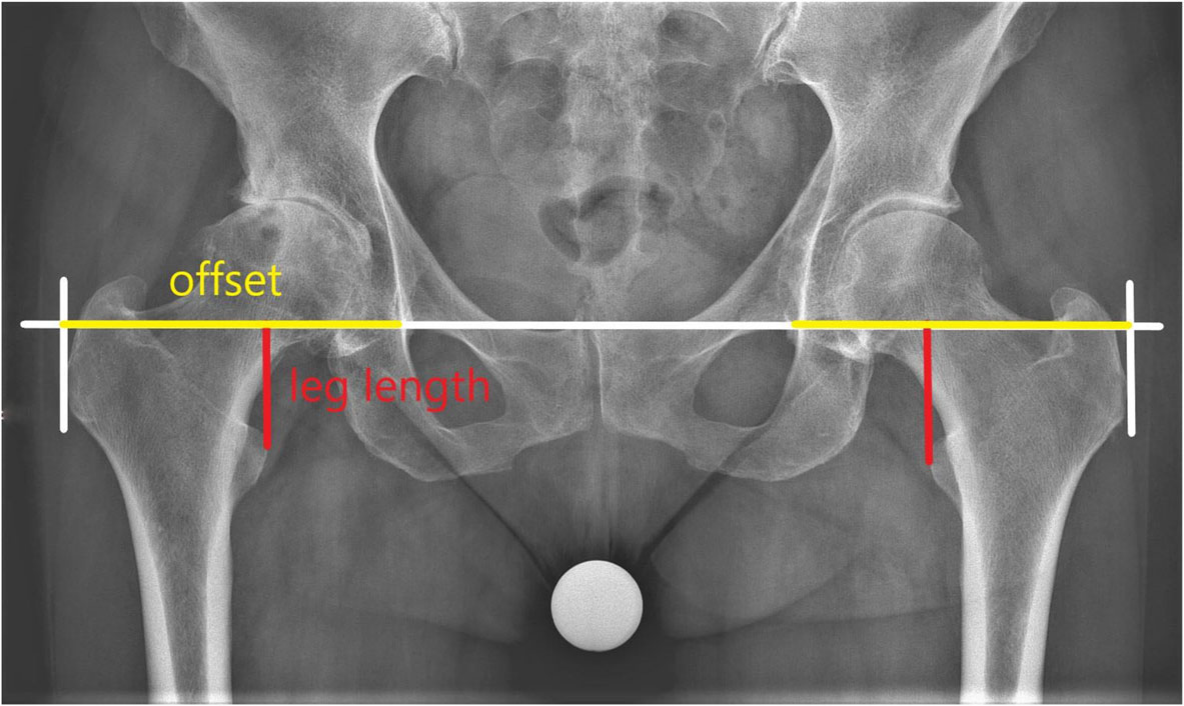
X-ray pelvis pre-OP

To avoid any bias, the radiological data were collected, examined, and evaluated completely independent by author MF. For each patient, a set of two radiological examinations, which were all performed at the same department using the same x-ray machine, was evaluated. An anterior-posterior (AP) weight-bearing x-ray of the pelvis with patella facing forward was taken. Using the Woolson method [3], leg length was measured by drawing a line from the interteardrop line to the apex of the trochanter minor. The offset, which means global offset, was assimilated by a line, parallel to the interteardrop line, from the bottom of the teardrop to the height of the most lateral point of the trochanter major (Fig. 2).

According to this preoperative x-ray, a rough estimation of the required change in leg length and offset was calculated comparing the situation with the contralateral hip. At this point, also other possible pathologies, e.g., a future operation on the contralateral side, were considered. At our institution, we do not perform simultaneous bilateral THA. The perception of the likelihood of a second operation on the contralateral side and the consequences on the LLD of the second operation influenced our target of LLD of the first operation.

**Fig. 3:**
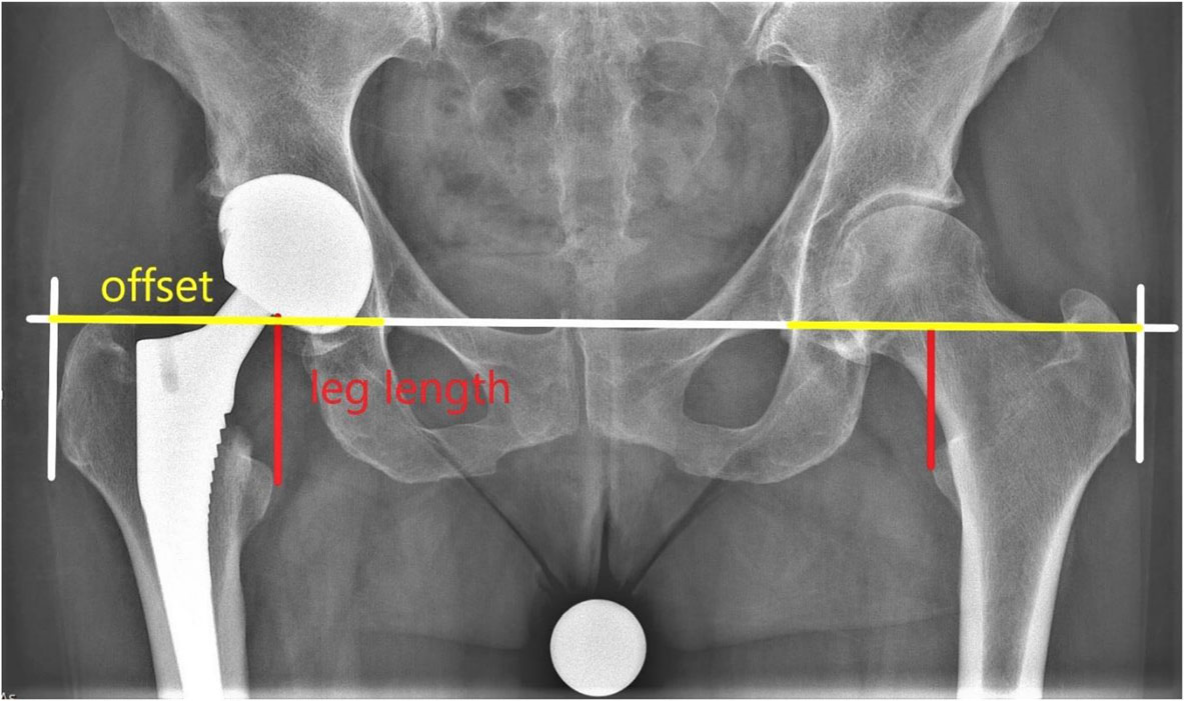
X-ray pelvis post-OP

Postoperatively, all patients were encouraged to be mobilized with full weight-bearing as tolerated. Dependent upon early mobilization and the patient’s pain level, a second x-ray of the pelvis was taken on the third postoperative day using the same technique (Fig. 3). A calibration ball of 25 mm was used for both examinations. It was placed between the patient’s legs as close to the focal point of the x-ray beam as practically possible.

### Statistics

Mean, median, range, and standard deviation were calculated for the various measurement parameters. Data was analyzed with the SPSS V22.0 statistical software (IBM Corp., USA). Pearson correlation testing was performed to compare the radiographic results with the intraoperative measurements of the change in leg length and the change in offset. This study hypothesized that these two values correlate, and herewith, the use of CAL predicts the postoperative leg length and offset. A correlation value of r ≥ 0.5 was found to be a strong correlation. A p < 0.05 was considered to be statistically significant.

No control group was available as CAL is used in all THA operations by the senior author.

## Results

Fifty-four patients and 59 hips with complete data sets were included for analysis. The mean age of the patients was 51 years (17–74), with 30 out of 54 patients being male. Fifty-six out of 59 operations were primary THA’s, and 3 were revisions. The indication for operation was degenerative osteoarthritis in 32 cases, rheumatoid arthritis 4 cases, hip dysplasia (Crowe I to III) 6 cases, and stage 4 non-traumatic avascular caput necrosis 10 cases. Four patients were operated because of posttraumatic avascular necrosis. The three revisions were performed due to polyethylene wear, aseptic loosening of a cemented acetabular component, and undercoverage of acetabular component, each. The BMI of the patients was mean 30 (20–47). The operational time was mean 127 min (78–211). When operated, 11 of the patients had already a THA on the contralateral side. The day of discharge was postoperatively mean day 4.8 (1–10). The use of the CAL increased the operational time by about 4 min. There were no intraoperative or postoperative complications reported due to the use of the CAL, especially no wound healing problems of the additional small proximal incision. On these 59 THA operations, the following complications were observed: on five patients, there was intraoperatively a suspicion of a proximal femoral calcar fracture which was treated by a cerclage. Two patients had more than 3° varus/valgus malalignment of the femoral stem. One patient suffered an undisplaced pelvic fracture intraoperatively and was treated conservatively without any further consequences. One patient had directly postoperatively an inlay displacement of the ceramic acetabular inlay; one patient had an undercoverage of the acetabular component. Both of these patients were taken for revision. Six patients had blood transfusion postoperatively.

The preoperative x-ray measurements show an LLD mean of −6.0 mm (−29 to 25), while the postoperative x-ray measurements show an LLD mean of 3.6 mm (−7 to 43). Of the operated 59 hips, 43 had an LLD up to 5 mm, 54 up to 10 mm, 57 up to 15 mm, and 2 over 20 mm, directly after surgery. Comparing the pre- and post-operative x-ray showed an increase in leg length on the operated side with a mean of 10.1 mm (−3 to 26), while intraoperatively on the calliper, a change of mean 10.0 mm (−3 to 27) was observed. This shows a high positive correlation of r = 0.873, p<0.01. Comparing the pre- and post-OP x-rays showed a decrease in total offset on the operated side of mean −3.68 mm (−18 to 11), while intraoperatively on the calliper there was observed change of mean −1.86 mm (−13 to 20). This also shows a strong correlation of r = 0.542, p<0.01. The results are summarized in Table 2.

**Table 2:** Pre-, intra- and postoperative measurements by x-ray and by CAL

	Mean (range) (mm)	SD	r	p
Pre-OP LLD	−6.00 (−29–25)	8.81		
Post-OP LLD	3.64 (−7–43)	7.29		
Change in LL				
Change as measured by CAL	10.01 (−3–27)	5.87		
Change as measured on x-ray	10.11 (−3–26)	6.28	0.873	p<0.01
Change in offset				
Change as measured by CAL	−1.86 (−13–20)	5.96		
Change as measured on x-ray	−3.68 (−18–11)	5.98	0.542	p<0.01

## Discussion

The final results on LLD of our patients post THA on the post-OP x-ray are comparable to the results of other studies using a CAL intraoperatively [11, 12]. There also is a good correlation of the intraoperative measurements of the leg length and total offset using the calliper in reference to the values measured on the anteroposterior x-ray of the pelvis. As shown in the literature, the use of a calliper intraoperatively significantly improves the results on leg length and offset in comparison to just using clinical tests or templating [11–13]. Using the same operative technique and approach, Wayne et al. measured on 100 primary THA postoperatively an LLD of mean 5.1 mm, without the use of a calliper [14]. The patients of this study reached a mean LLD of 3.6 mm, with the use of a calliper. Similarly with all other studies, we also considered the following targets: no leg length discrepancy and the restoration of the offset on the x-ray pelvis taken postoperatively. Of the different existing methods to measure LLD on the x-ray pelvis, we used the Woolson method [3], which also is associated with 4–5 mm error in measurements [15]. As mentioned, additional clinical tests to check stability and impingement were done intraoperatively. There were no patients where it was necessary to change the neck length or other prosthesis parts to prioritize stability over achieving the scheduled limb length and offset, abandoning the calliper input. In agreement with Barbier et al., the calliper seems to better predict the leg length than the offset [12]. Our study also confirms that good results using the calliper can be achieved with other surgical approaches as we used a different approach than Barbier et al. or Enke et al. [11, 12].

In his two keystone publications on LLD, Gurney et al. concluded that it is generally challenging to find a perceptive, functional, and anatomic patient treatment benchmark for LLD [4, 16]. Options to treat LLD in adults include physiotherapy-guided exercises, modifiable heel lift, or post-surgical interventions [4]. Although there might be a break point around an LLD of 20 mm, every patient presenting an LLD needs to be considered individually on a case-by-case basis. Patient communication and education should include discussing the different treatment options. There was no incidence of return to surgery for revision with any patients included within this study related to postoperative LLD.

Among our patients were several with severe pelvis pathology or posttraumatic avascular caput necrosis presenting preoperatively as an LLD of around −2.9 cm, for the leg to be operated shorter than the contralateral side. Excessive limb lengthening increases the odds for nerve palsy [17]. Therefore, our target of reaching complete correction of LLD was compromised. In these cases, even experienced hip surgeons find it difficult to evaluate the amount of increase of LL intraoperatively [18]. We found the calliper to be especially useful during operations when facing these challenges.

There are different CALs on the market, using the same principle with a fixed reference point on the pelvis and a mobile point on the femur [7, 11]. They all are similar and function successfully. This study utilized the CAL from Smith & Nephew because of our long-lasting experience with it. It is not cost-prohibitive in relation to instrument supply and has reliable international availability. The literature and the results of our study show convincingly that the use of an intraoperative calibration gauge improves significantly the postoperative results concerning LLD [6, 11, 12].

Within the literature, other authors have expressed doubts that this is also the case concerning the offset [12]. One apparent reason is the fact that the calliper measurements are related to a change in “total offset” and not in “femoral offset,” which seems to be more important for the functional outcome after THA [5, 19]. We compared the postoperative total offset with the preoperative offset of the same hip. In the literature, there is disagreement about which value should be used as reference when comparing postoperative offset after THA. Some authors use as reference the preoperative offset of the operated hip [5, 20], some use the offset of the contralateral not operated hip [19], while others use absolute values as 42–48 mm [21].

Similar to some studies, we chose to measure the change of offset on digital radiographs of the pelvis [11, 12, 20]. We consider this a major weakness of this study. Reproducibility is poor, with a mean error of about 9.7 mm, which means 22% assuming an offset of 45 mm, when using x-ray for measuring offset [22]. While CT seems to be the golden standard to measure offset [23], already a software upgrade of the digital x-ray gives more precise results Ein-Bild-Röntgen-Analyse [5]. Due to software incompatibility with our existing PACS system, this upgrade was not used for this study.

Although interest in postoperative offset after THA is increasing, it is still unconfirmed that differences in offset lead to severe complications. In a meta-analysis study, De Fine et al. did not report any difference in bearing surface wear, implant loosening, or dislocation rate when looking at THA patients with different femoral offsets using hard-bearing surfaces [24]. A study from the New Zealand registry does not show any difference in the revision rate related to different offsets due to different designs of uncemented femoral stems [21]. Studies investigating clinical results after THA report the growing tendency that patients with decreased femoral offset are more Trendelenburg positive and have a worse outcome on the Oxford Hip Score (OHS) than patients with a restored or increased offset [5, 10, 25, 26]. This might emphasize the importance of restoring the abductor lever arm to at least a certain length [19]. Summarizing these results, in case of indecision rather an increase in offset might be recommended [5, 10, 19].

In our study, we saw a strong correlation between the results of our intraoperatively used calibration gauge with the measurements on the x-ray pelvis concerning change of offset r = 0.542, p<0.01. Despite reviewing available literature, we still have challenges with a proper interpretation of these results in relation to the benefit for the patient. We cannot even define a clear target zone for postoperative offset. In the clinical setup, any LLD seems to be more relevant than any discrepancy in offset. Therefore, when intraoperatively a situation arises where a mismatch in LL and offset has to be compromised, the LL should be made accurate. Further studies will be required to clarify this situation.

Postoperative migration and subsidence of both components, the acetabular and the femoral component, are known and well-studied phenomena after THA surgery. The Corail stem, used for 57 of our 59 operated hips, is uncemented and fully hydroxyapatite coated. According to Selvaratnam et al., most of the subsidence of this stem occurs within the first 6 weeks after the operation [27]. In literature, the amount of postoperative subsidence of the stem and the percentage of patients concerned with it greatly differ. Faisal et al. report that the uncemented collarless Corail stem can be used safely for all patient groups, even the elderly, and almost no subsidence occurs [28]. Using the same stem, Ries et al. found a mean subsidence of 3.1 mm after a mean follow-up of 7 months [29]. Other authors report that about 30% of the stems subside more than 3 mm within the first 6 weeks [27, 30]. It might be considered that the use of a collared stem is the answer to avoid stem subsidence. However, in clinical series, only about 39% of collars have primary contact to the bone [31]. In a large series, it is shown that the mean subsidence of uncemented stems is 2.9 mm (0–20.4 mm) whereas the addition of a collar leads to a lesser degree of subsidence but does not avoid it [29]. A collarless uncemented stem respects the press-fit principle and, therefore, should lead to better bony osseointegration of the prosthesis and less aseptic loosening. It should be mentioned that even a cemented femoral stem, the Exeter stem, has a subsidence of 1.42 mm (0.43–3.91) within the first 2 years and afterwards 0.08 mm/year [32].

The Pinnacle cup, used for 57 of our 59 operated hips, is uncemented, hemispheric, and fully hydroxyapatite coated. According to Dammerer et al., within the first 2 years, this cup shows a mean total migration of 1.42 mm (0.1–6.3) [33]. Another study using a similar cup shows an even higher migration within the first 2 years postoperatively for patients being operated for rheumatoid arthritis 2.62 mm (0.55–8.22) than for osteoarthritis 1.44 mm (0.1–5.62) [34]. Nieuwenhuijse et al. report that even the cemented Exeter cup shows a mean cranial migration of 0.94 mm within the first 2 years [35].

Until now, all studies evaluating leg length after THA are based on the target that, directly postoperatively, the patient should have the same leg length as the contralateral side [36]. The patients’ expectations are set up the same way. Even though the leg length postoperatively might be precisely the same, the patient often has a feeling immediately after the operation that the operated extremity is “too long”. This may be due to postoperative swelling and pain and usually disappears within the first weeks. Another study reveals that even at the long view, 64% of patients after THA perceive an LLD despite radiologically this is not apparent, setting the threshold for LLD at ≥ 5 mm [1]. The list of states that can lead to a perception of LLD is long: spine pathology, pelvic obliquity, LLD concerning status post THA, neurological impairment, knee malalignment [37], and other discrepancies below the hip, leading to a very complex situation. According to the authors, there is an additional time-dependent factor to be considered: the migration of the prosthesis components, which contributes to a change in perception of LLD. Summarizing the studies named above, patients can easily expect a change in LLD of 5 mm or more sometime after surgery due to subsidence of the stem and the cup. Subsidence of prosthesis components after THA is common. A certain amount of subsidence and long-term shortening of the extremity should not be evaluated as a complication. It should be seen as the prosthesis’ natural process when embedded in a living environment. In our opinion, the orthopedic surgeon, the patients, and legal institutions have to consider these factors when evaluating the success of a THA operation. This must be reflected upon intensively in pre- and postoperative counseling and be part of the surgical consent of the THA patient.

## Conclusion

The technique using an intraoperative calibration gauge to predict postoperative leg length and total offset is safe and does not lead to any complications. It improves the postoperative results concerning LLD, and therefore, we recommend that the tool be used routinely for all THA operations. This study was done to describe the technique and to establish its use. Further comparative controlled studies will be needed to confirm the advantage of this technique. The intraoperative gauge gives also good estimation of postoperative offset, but its clinical and anatomical relevance is not clear and should be subject to further studies. We suggest a target of +5 mm of direct postoperative LLD after consideration of the frequency and consequences of subsidence of the prosthesis components of THA patients.

## Data Availability

Herewith, the authors confirm that all data and materials, used for this study, are available on request.

## Abbreviations

BMI: Body mass index
CAL: Calliper
CT: Computed tomography
F: Female
L: Left
LL: Leg length
LLD: Leg length discrepancy
M: Male
OHS: Oxford hip score
OP: Operation
PACS: Picture Archiving and Communications Systems
R: Right
THA: Total hip arthroplasty
TN: Tennessee
UAE: United Arabic Emirates
USA: United States of America
